# Investigation of the Feasibility of Ventricular Delivery of Resveratrol to the Microelectrode Tissue Interface

**DOI:** 10.3390/mi12121446

**Published:** 2021-11-25

**Authors:** Youjoung Kim, Evon S. Ereifej, William E. Schwartzman, Seth M. Meade, Keying Chen, Jacob Rayyan, He Feng, Varoon Aluri, Natalie N. Mueller, Raman Bhambra, Sahaj Bhambra, Dawn M. Taylor, Jeffrey R. Capadona

**Affiliations:** 1 Department of Biomedical Engineering, Case Western Reserve University, Cleveland, OH 44106, USA; 2 Advanced Platform Technology Center, Louis Stokes Cleveland Veterans Affairs Medical Center, Cleveland, OH 44106, USA; 3 Veteran Affairs Ann Arbor Healthcare System, Ann Arbor, MI 48105, USA; 4 Department of Biomedical Engineering, University of Michigan, Ann Arbor, MI 48109, USA; 5 Department of Neurology, University of Michigan, Ann Arbor, MI 48109, USA; 6 Cleveland Functional Electrical Stimulation Center, Louis Stokes Cleveland Veterans Affairs Medical Center, Rehabilitation Research and Development, Cleveland, OH 44106, USA; 7 Department of Neurosciences, Cleveland Clinic Lerner Research Institute, Cleveland, OH 44195, USA

**Keywords:** intracortical microelectrode, antioxidant, ventricular drug delivery, neural, neural recording, foreign body response

## Abstract

(1) Background: Intracortical microelectrodes (IMEs) are essential to basic brain research and clinical brain–machine interfacing applications. However, the foreign body response to IMEs results in chronic inflammation and an increase in levels of reactive oxygen and nitrogen species (ROS/RNS). The current study builds on our previous work, by testing a new delivery method of a promising antioxidant as a means of extending intracortical microelectrodes performance. While resveratrol has shown efficacy in improving tissue response, chronic delivery has proven difficult because of its low solubility in water and low bioavailability due to extensive first pass metabolism. (2) Methods: Investigation of an intraventricular delivery of resveratrol in rats was performed herein to circumvent bioavailability hurdles of resveratrol delivery to the brain. (3) Results: Intraventricular delivery of resveratrol in rats delivered resveratrol to the electrode interface. However, intraventricular delivery did not have a significant impact on electrophysiological recordings over the six-week study. Histological findings indicated that rats receiving intraventricular delivery of resveratrol had a decrease of oxidative stress, yet other biomarkers of inflammation were found to be not significantly different from control groups. However, investigation of the bioavailability of resveratrol indicated a decrease in resveratrol accumulation in the brain with time coupled with inconsistent drug elution from the cannulas. Further inspection showed that there may be tissue or cellular debris clogging the cannulas, resulting in variable elution, which may have impacted the results of the study. (4) Conclusions: These results indicate that the intraventricular delivery approach described herein needs further optimization, or may not be well suited for this application.

## 1. Introduction

Intracortical microelectrode have been used for decades in basic brain research and are a critical part of neural interfacing systems designed to improve the quality of life for those with amputations, spinal cord injury, or degenerative brain diseases [1,2]. Early work in microelectrodes was mainly for basic science to elucidate the mystery of single neurons and their activities in the brain, using Ag/AgCl based electrodes [3]. The electrodes have steadily evolved to include various materials and designs to improve biocompatibility and performance, as well as processing methods to improve neuronal activity acquisition and analysis [4,5,6,7,8,9]. Ultimately, the advancement of microelectrode technology has allowed for current applications such as brain mapping, neural prosthetics use for the recovery of lost mobility and sensations, and the treatment of neurodegenerative diseases such as Parkinson’s disease [2]. Unfortunately, IMEs have been shown to fail over time, decreasing their potential for long-term basic science or clinical applications [10,11]. Failure of IMEs is often characterized by increasing noise and decreasing recorded signal amplitudes resulting in a declining signal-to-noise ratio (SNR) over time. This declining SNR results in fewer channels detecting single units and fewer single units detected per channel [12]. There are several proposed mechanisms of intracortical microelectrode failure, including mechanical breakage, biological inflammatory response, and material breakdown [10]. Of the failure mechanisms, oxidative stress is implicated in both the biological inflammatory response and the material breakdown—specifically exacerbating the foreign body response of immune cells, and damaging the insulating and conducting layers of the probe itself [13,14]. As a result, drug delivery of antioxidants and other anti-inflammatory drugs have been explored as a way to decrease oxidative stress and improve the performance and lifetime of microelectrodes. Unfortunately, due to limiting factors such as low solubility, metabolism, and the blood–brain barrier, delivery of adequate therapeutic concentrations of drugs have proven difficult. In this paper, we explored the use of an intraventricular cannula delivery of resveratrol, an antioxidant, and its efficacy to deliver to the site of affected tissue.

The implantation of the electrodes into the brain results in the breaching of the blood–brain barrier, thereby releasing blood proteins and other proinflammatory factors into the parenchyma [11,15,16,17]. The insults from device implantation leads to the activation of microglia, infiltrating macrophages, and astrocytes [15,17]. The phenotypic change of these cells to an activated inflammatory state results in the upregulation of inflammatory cytokines, chemokines, and ROS/RNS. Chemokines and cytokines attract and activate more microglia and infiltrating macrophages to the injury site, which in turn release more soluble inflammatory factors [15,18]. Activated microglia begin to upregulate (nicotinamide adenine dinucleotide phosphate (NADPH) oxidase, which leads to elevated ROS/RNS levels [19,20]. Endogenous enzymes such as superoxide dismutase (SOD) and catalase that regulate ROS/RNS levels are unable to clear the increased levels of ROS/RNS near the implant site. Increased ROS/NOS concentrations may damage surrounding structures and tissue via oxidation and peroxidation of lipids, proteins, and DNA [14]. Damage from ROS/RNS may lead to activation of cell death pathways, leading to decreased survival of neurons near the implant site [14,20,21]. Additionally, activated astrocytes migrate to the implant site in order to isolate the IME, a foreign body, from the rest of the healthy brain tissue by building a glial scar, thereby creating a barrier that prevents close proximity of neurons and further hinders recording quality [22,23,24]. Unfortunately, accurate single unit activity recording requires neurons to be within a 140 μm distance from the electrode [25].

ROS/RNS play a large part in further exacerbating oxidative stress as well as negatively impacting both tissue health and microelectrode integrity. Exogenous antioxidant administration has been proposed by researchers as a method of complementing endogenous antioxidant enzymes to reduce the amount of ROS/RNS available or generated, thus decreasing oxidative stress and the foreign body response [26,27,28]. Polyphenol antioxidants such as curcumin and resveratrol have shown properties such as neuro-protection and anti-proliferation in cancer, and have aided in decreasing the inflammatory response to injury [26,29,30,31,32,33,34]. Such antioxidants directly scavenge ROS/RNS species and their precursors to decrease levels of ROS/RNS, and initiate the upregulation of endogenous antioxidant enzymes [26]. Our lab has previously explored acute intraperitoneal (IP) administration of resveratrol on the day before and the day of microelectrode implantation. We found that delivery of resveratrol resulted in a decrease of ROS levels, blood-brain barrier permeability, and neuronal nuclei density near the implant site at two weeks [13]. This positive result led to a chronic study with daily IP administration of resveratrol, which showed oxidative stress mitigation and decreased neuronal degeneration up to two weeks post-implantation. After 16 weeks, there was also decreased neuronal degeneration near the implant in the group receiving resveratrol compared to controls. Although the daily injections in that study resulted in sustained therapeutic resveratrol concentrations around the implant site, the repeated IP injections led to scar tissue and adhesions in the abdomen [35]. Due to the low solubility of resveratrol in aqueous solutions (i.e., saline) and low bioavailability attributed to first pass metabolism, delivering adequate concentrations of resveratrol to the brain was a challenge [29,36,37,38]. Therefore, investigation of localized delivery methods was performed by Nguyen et al. by doping a compliant nanocomposite electrode with resveratrol during fabrication [39]. After two weeks, Nguyen et al. found decreased activation of microglia and macrophages and an increased neuronal nuclei density near the implant. However, at 16 weeks post implantation, there was no difference in tissue response due to depletion of the loaded drug at the chronic time point. There were improvements in tissue response shown in the presence of local resveratrol delivery, yet no therapeutic effects were observed when resveratrol was exhausted. Additionally, the initial studies with localized resveratrol delivery utilized non-functional electrodes, lacking the important correlation between the inflammatory response to the implant and electrode recording performance.

Here, we present the implementation of an intraventricular delivery system for long-term, controlled, and sustained delivery of resveratrol directly to the brain. The current study aimed to explore the efficiency of resveratrol delivery through the ventricular system to reach the site of implanted IME. Furthermore, electrode performance and tissue response were also evaluated. Intraventricular delivery circumvents the blood–brain barrier and first pass metabolism, requiring decreased dosing for increased therapeutic effect. Electrode performance was evaluated for six weeks, as this was the longest acting pump available from the manufacturer (Alzet Cupertino, Cupertino, CA, USA), and represents an appropriate time scale for tissue healing to occur. Post-mortem histological assessment of inflammation and oxidative stress was also compared to control animals. The concentration of resveratrol around the electrode was quantified using mass spectrometry to confirm adequate concentrations were being delivered.

## 2. Materials and Methods

### 2.1. Alzet Osmotic Pump Assembly

In order to ensure delivery of resveratrol to the parenchymal tissue near the electrode, Alzet brain infusion kits and osmotic pumps (models 1 and 2006 respectively; Alzet, Cupertino, CA, USA) were utilized for intraventricular delivery. Brain infusion kits and osmotic pumps were assembled per manufacturer’s protocol. In a sterile biosafety hood lined with sterile drapes, the stainless steel, 28 gauge cannula was adjusted to a penetration depth of 3.5 mm with the addition of the included spacers. This standardized the depth of implantation of the cannula to assure it would reach the lateral cerebral ventricle. The characterization to validate the penetration depth as well as confirm the coordinates of the lateral cerebral ventricle were performed by implanting a brain kit and osmotic pump filled with food coloring (data not shown). Next, the catheter tubing was cut to 5 cm in length, allowing enough space to attach the cannula to the osmotic pump that was implanted subcutaneously in the back. The catheter tube was then flushed with sterile saline, and one end of the tube was attached to the cannula while the other end was attached to the flow moderator, which would be later inserted into the pump reservoir. Autoclaved pure trans-resveratrol powder (Mega Resveratrol, Danbury, CT, USA) was dissolved in filter sterilized Poly(ethylene glycol) (PEG; MW200, Sigma-Aldrich, St. Louis, MO, USA) in a sterile hood for a final concentration of 500 mM of resveratrol in PEG200. Using a sterile syringe, 200 µL of the sterile 500 mM resveratrol/PEG200 solution was filled into each of the osmotic pump reservoirs. The flow modulator was attached to the osmotic pump and the fully assembled cannula/osmotic pumps were primed in a sealed sterile container filled with sterile saline for 60 h at 37 °C. At the end of priming, the cannula/osmotic pumps were implanted into the animal subjects following the procedure detailed below.

### 2.2. Intracortical Microelectrode and Cannula Implantation Procedure

All animal procedures were performed as approved by the Institutional Animal Care and Use Committee (IACUC) at the Louis Stokes Cleveland Department of Veterans Affairs Medical Center and all experiments were performed in accordance with relevant guidelines and regulations. The impedance values of NeuroNexus 16 channel, silicon, single shank intracortical microelectrodes (NeuroNexus, Anne Arbor, MI, USA, A1 × 16-3 mm-100-177-Z16) were verified with the manufacturer’s values before implantation by measuring the electrode impedance at 1 kHz in saline, and then cleaned in 95% ethanol and deionized water for five minutes each. All probes were then sterilized using ethylene oxide gas at 12.2 °C for 1 h, and aerated for 12 h, following a standard protocol previously used in our laboratory [14]. Two groups of Sprague Dawley rats (Charles River, Wilmington, MA, 11 weeks old, ~250 g, N = 8 per group) had sterilized electrodes implanted in their primary motor cortex. The resveratrol group also received an infusion pump with the delivery cannula in the ventricle and the reservoir in the animal’s back.

The surgical protocol was similar to previously published methods [40]. Briefly, rats were placed in an isoflurane chamber flowing 3% isoflurane and 1.5 L/min O_2_, until anesthetic plane was confirmed with a toe pinch. Once anesthetic plane was confirmed, the rats were mounted in a stereotaxic frame under isoflurane flowing 2–2.5% isoflurane and 1.5 L/min O_2_ and provided Carprofen (5 mg/kg) and Cefazolin (25 mg/kg), along with Marcaine (0.25%) at the surgical site. Vitals were monitored using a MouseSTAT Pulse Oximeter and Heart Rate Monitor (Kent Scientific Corp., Torrington, CT, USA), which also included a regulated heating pad placed under the animal to maintain body temperature.

After the initial midline incision on the scalp, the skin was retracted to expose the skull, and periosteum was cleaned off using a cotton tip applicator. Hydrogen peroxide and Vetbond (3M, St. Paul, MN, USA) were applied to first dehydrate, and then prime the skull for craniotomy. Craniotomies were made using a Kopf dental drill (David Kopf Instruments, Tujunga, CA, USA) using a 1 mm drill bit, alternating between drilling and a saline irrigation to minimize thermal damage to the blood–brain barrier [41]. The resveratrol group received a craniotomy over the lateral ventricle for the cannula at 1 mm posterior to bregma and 1.5 mm lateral to midline. The cannula was implanted and held in place with a small amount of Vetbond and Teets Cold Cure Dental Cement (A-M Systems, Sequim, WA, USA). A subcutaneous pocket was created in the back of the animal and the osmotic pump was inserted. The manufacturer specifications for the osmotic pumps used in this study stated a continuous delivery of 0.13 µL/h (356.07 µg resveratrol/day) for 6 weeks. Additional craniotomies for the ground (1.5 mm lateral to midline and 1.5 mm posterior to bregma) and reference wires (1.5 mm lateral to midline and 5.5 mm posterior to bregma) were made in the opposite hemisphere in both groups. The respective ground and reference wires of the microelectrode were lowered by hand into the brain to ~2 mm in depth and held in place with a small amount of dental cement.

**Figure 1:**
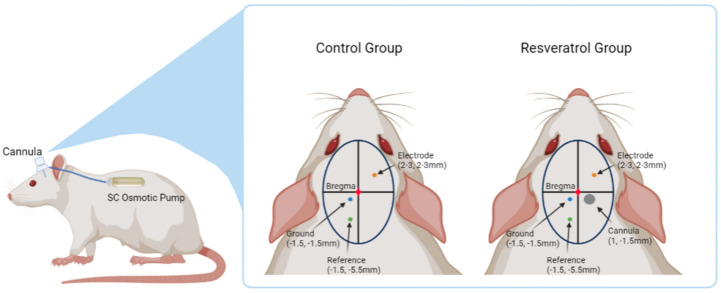
Illustration of the placement of all implanted components. The electrode (2–3 mm anterior and 2–3 mm lateral to bregma), ground (1.5 posterior and 1.5 lateral to bregma), and reference (5.5 posterior and 1.5 lateral to bregma) were implanted at the same coordinates for both the control and resveratrol group. The resveratrol group was additionally implanted with the cannula at 1 mm posterior and 1.5 mm lateral to bregma. A subcutaneous pocket was made in the animal’s back to place to osmotic pump. Created with Biorender.com.

A final craniotomy was made over the forelimb primary motor cortex and the dura reflected for implantation of the microelectrode. The electrode was held using a micromanipulator attached to the stereotaxic frame, and lowered slowly into the primary motor cortex within an area spanning 2–3 mm lateral to the midline and 2–3 mm anterior to bregma. The specific location was chosen for each rat to avoid surface vessels and to avoid hitting the infusion cannula in the treatment group. The electrode was inserted 100 µm every 1 to 2 min to a depth of ~1900 µm. The neural signal was monitored and acute recordings were performed under anesthesia during the implantation process, using Synapse software from Tucker Davis Technologies, to confirm the 16 channels spanned the cell-dense layers (i.e., Layers III and V). Kwik-Cast (World Precision Instruments, Sarasota, FL, USA) was applied to seal the craniotomy, and a headcap of dental cement was made around the electrode connector to secure the cap in place. The skin was then sutured closed over the head cap using 5-0 monofilament polypropylene sutures (Henry Schein, Melville, NY, USA), leaving enough space to allow the Z16 head stage (Tucker-Davis Technologies, Alachua, FL, USA, Catalog #ZC16) to be clipped to the connector. All animals were monitored for 5 days post operation to ensure proper healing, and both analgesics (Carprofen 5 mg/kg once a day for 2 days), and antibiotics (Cefazolin 25 mg/kg twice a day for 3 days) were administered during this time. Figure 1 illustrates the placement of all components implanted into the animals for both the control group and resveratrol group.

### 2.3. Electrophysiological Recordings

Electrophysiological recordings were taken after regaining consciousness from surgery and the day after surgery. Subsequently, electrophysiology was recorded from each animal twice a week for the entire 6-week study duration. In brief, animals were placed in a chamber flowing 3% isoflurane in 1.5 L/min O_2_ for 2 min to limit animal movement while the head stage was connected to their implants to ensure the head cap was not damaged during connection. Animals were then allowed to fully wake up until sternal and walking with a normal gait in an open box before starting 10 min of continuous recording. A TDT RZ5D BioAmp Processor recording system (Tucker-Davis Technologies, Alachua, FL, USA) set at 24,414 Hz sampling rate was used.

### 2.4. Signal Processing

Neural signal recordings were processed as previously described using custom MATLAB scripts [40]. Specifically, the 24,414 Hz sampled data was bandpass filtered between of 300–3000 Hz and common average referenced to remove environmental and system noise common to all channels before further processing. Visible artifacts (e.g., due to cables hitting the cage sides, chewing artifacts, etc.) were then removed manually by a reviewer blinded to both the animal ID and time point. The standard deviation of the background noise was robustly estimated as the median of the absolute deviation of the raw detected voltage divided by 0.6745 [42]. Two times this value was considered the noise amplitude for SNR calculations. Spikes were detected if the voltage crossed a threshold 3.5 times the standard deviation estimate. Signal to noise ratio could then be calculated using the peak-to-peak amplitude of the mean spike waveforms divided by the noise estimate for each channel. Wave_clus software was used for sorting detected spikes into multiple single units (minimum spikes required to be considered a single unit was set to 20) [42]. To account for some inherent variance in implantation depth between animals, performance metrics were calculated using the best ten consecutive channels with the assumption these channels spanned the cell-dense layers of III and V. The following metrics were calculated from those ten channels: number of detectable single units per channel (units per channel), percent of channels able to detect a single unit (SU Yield), average signal amplitude from channels detecting signals, average background noise, and average signal to noise ratios (SNR). The definition of a “unit” in this study is a recorded neuron that had an action potential with a distinct wave shape that could be separated from the background noise. Individual days or channels were removed from analysis if there were problems obtaining accurate recording data for that day as a result of debris in the connector, improper headstage connections, or other complications. Removed days and channels consisted of 1.79% of recording days and only 0.07% of channels throughout the course of the study.

### 2.5. Tissue Processing

Animals were anesthetized with an IP injection of Ketamine (160 mg/kg) and Xylazine (20 mg/kg) at the pre-determined end point of the study. Anesthetic plane was monitored with a toe pinch and animal movement. When the animal was sufficiently anesthetized, a lateral incision through the skin and abdominal wall under the rib cage was made. The diaphragm was cut along the entire length of the rib cage carefully to expose the pleural cavity, and the ribs were cut from the base to the collarbone on both sides. The sternum was clamped with a hemostat to hold it above the head to ensure a clear view of the heart and the major blood vessels. A small incision was made to the left ventricle and the gavage was inserted until it reached the aorta. When the end of the gavage was visible in the aorta, another hemostat was used to clamp it in place and the right atrium was cut to allow blood and perfused liquids to flow out. The rat was perfused with 400–500 mL 1X phosphate buffer saline (1X PBS, Invitrogen, Calsbad, CA, USA) until the fluid flowing out of the right atrium was clear and the liver was a beige color. Following the first solution, 200–300 mL of 30% sucrose solution (30% sucrose by weight in 1X PBS) was perfused. Following this, the brain was harvested from the skull, soaked in 30% sucrose for two days, and then frozen in optimal cutting temperature compound (Tissue Tek, Torrance, CA, USA) by placing it in an insulated container of dry ice, and then further stored at −80 °C until cryosectioning. Brains were sliced transversely into 20 µm thick slices and mounted on glass slides for use in fluorescent immunohistochemistry.

### 2.6. Immunohistochemical Staining

Standard immunohistochemistry protocols from our lab were utilized for the assessment of activated microglia/macrophages, astrocytes, blood-brain barrier permeability, and neuronal density around the electrode interface [14,39]. Tissue was thawed at room temperature in a humidity chamber for one hour, followed by gentle application of 1XPBS with plastic transfer pipettes to rehydrate the tissue slices. Subsequently, tissue was fixed with 4% paraformaldehyde for 15 min before being permeabilized for 15 min in PBS-T (1X PBS, 0.1% Triton-X100) and blocked with goat serum blocking buffer (10% normal goat serum, 1XPBS, 0.1% Triton-X100, 0.01% sodium azide) and incubated at room temperature for 1 h. Rabbit anti-immunoglobulin G (IgG, 1:100, Millipore Sigma, Burlington, MA, USA, Catalog #AP124) was applied to the first batch of tissue slices overnight at 4 °C. The next day, mouse anti-neuronal nuclei (NeuN 1:250, Millipore Sigma, Burlington, MA, USA, Catalog #MAB3477) to visualize neuronal cells was applied to the same tissue slices overnight at 4 °C. A second batch of tissue slices were incubated with both rabbit anti-glial fibrillary acidic protein (GFAP, 1:500, Agilent Dako, Santa Clara, CA, USA, Catalog #Z0334429-2) to visualize astrocytes and mouse anti-CD68 (1:100, Millipore Sigma, Burlington MA, USA, Catalog #MAB1435) to visualize activated microglia and macrophages overnight at 4 °C. After application of primary antibodies, all of the tissue slices were washed with PBS-T and fluorescent AlexaFluor secondary antibodies 488/594 (1:1000, Invitrogen, Waltham, MA, USA, Catalog #A11029, and #A11037, respectively) were added to visualize markers along with 4′6-diamidino-2-phenylindole (DAPI) (1:3600, 10.9 mM, Thermo Fisher Scientific, Waltham, MA, USA, Catalog #D3571) to visualize cell nuclei for two hours at room temperature. Consecutively, tissue was washed with PBS-T and incubated in copper sulfate buffer (0.5 mM copper sulfate in ammonium acetate for 10 min) to reduce tissue autofluorescence [43]. Tissue was then washed with dH2O and finally mounted with Fluoromount-G before being left overnight to dry.

Additionally, histological stains to quantitatively evaluate the extent of oxidative stress (rabbit anti-nitrotyrosine 1:500 Cayman Chemical, Ann Arbor, MI, USA, Catalog #10189540) were performed loosely following previous methods [14,44]. After washing with 1XPBS-T (0.1% Triton X-100 in complete 1X PBS) post formaldehyde fixation, antigen retrieval was performed by incubation in 0.01M sodium citrate buffer (2.94 g tri-sodium citrate, 1L dH20, pH adjusted to 6.0) for 2 min at 93 °C~99 °C and under pressure (InstaPot Plus LUX60V3, Kanata, ON, Canada). Peroxidase block (3% H_2_O_2_, 10% methanol, in complete 1XPBS) was applied to block active aldehydes and reduce background non-specific binding. Following this, the secondary staining including DAPI staining was performed as described above.

The explanted cannulas of the resveratrol group at the end of the 6 week time point was scraped of any matter clogging the inside using a 29 gauge needle. The material on the needle was spread on a glass slide with a drop of deionized water and allowed to completely air dry. The cells were then fixed onto the glass slide using a drop of methanol followed by a drop of 4% formalin. The slides were then incubated with DAPI (1:3600, Thermo Fisher Scientific, Waltham, MA, USA, Catalog #D3571) for 15 min and washed off with three consecutive washes of deionized water. The slides were mounted with glass coverslips using Fluoromount-G and left to dry overnight.

### 2.7. Image Analysis

All fluorescent stains were imaged using an Axioscan.Z1 (Zeiss Inc., Oberkochen, Germany) at 20× objective under the same exposure times for each fluorescent marker. Holes in the tissue left by the implanted electrodes were identified. Images were then exported as 16-bit tagged imaging files (TIFs) and imported into a custom MATLAB script called SECOND wherein the electrode implant sites were outlined and artifacts excluded [14]. In SECOND, each fluorescent marker was subsequently quantified in 50 mm wide concentric rings, spanning out to 750 µm away from the implant site. Background fluorescent intensity was defined as the average intensity determined in the 700–750 µm distance bin, and used to normalize the intensity in all bins. Neurons were manually counted using a custom MATLAB script called AfterNeuN [14]. Background neuronal density was defined as the number of neurons residing in the 450–500 µm distance bins, and all bins within the 0–450 µm distance were normalized using this background density value.

Optical microscopy of the explanted cannulas of the resveratrol group were taken using a high magnification Keyence microscope (VHX-7000, Osaka, Japan) to observe their state post-explantation both inside and outside of the cannulas.

### 2.8. Bioavailability Surgery

Following the surgical procedures outlined in Section 2.2 above, a separate set of animals was implanted with cannulas and osmotic pumps to evaluate the bioavailability of resveratrol. This group of animals were implanted with 2 mm non-functional, single shank neural probes instead of functional neural probes since electrophysiology metrics would not be evaluated here. There were four animals per group (N = 4) for the following four time points: 1 week, 2 weeks, 4 weeks, and 6 weeks [45,46,47,48].

#### 2.8.1. Tissue Harvest and Osmotic Pump Explant

Animals used for the bioavailability study were euthanized in a CO_2_ chamber and decapitated at each of their respective time points to prevent the washing away of resveratrol and its metabolites during perfusions. Brain tissue samples were collected using a 2 mm biopsy punch from the implant site and the hemisphere contralateral to the implant site from harvested brains. Biopsy samples from harvested livers were also collected to evaluate bio-distribution of resveratrol. In addition to the biopsy samples from the experimental animals, naïve rats that were not administered any drugs or treatment were sacrificed and biopsy samples from both hemispheres of the brain and liver were taken to serve as a blank reference for mass spectrometry analysis.

In order to ensure constant drug flow through the cannula connected to the osmotic pump during implantation, the actual flow rates observed were compared with the manufacturer’s predicted flow rate for the specific lot. Before implantation, the osmotic pumps (N = 4 per group) were weighed before and after filling to confirm the volume of solution in each pump reservoir. After animals were sacrificed and decapitated, the osmotic pumps were explanted and the remaining drug solution inside was extracted using a syringe and needle. The solution was then transferred to a micro-centrifuge tube, where a micropipette was used to draw up the solution to determine the remaining volume. The amount of drug solution remaining was subtracted from the amount of starting drug solution to determine the amount of drug solution eluted from the pump. These values were collected from the four different time points evaluated and plotted via a linear fit against the expected flow rate per manufacturer.

#### 2.8.2. Liquid Chromatography-Mass Spectrometry (LC-MS) Analysis

The biopsy tissue samples collected from the rats at each time point (N = 4) were put into micro-centrifuge tubes and frozen using dry ice. The samples were then shipped to PhenoSwitch Bioscience (Sherbrooke, QC, Canada), where the mass spectrometry analysis was done. All of the tissue samples, including the reference blank samples were processed similarly. Once at the facility, the samples were weighed before being transferred into 2 mL Eppendorf tubes containing 2.8 mm ceramic beads. Then 500 mL of extraction solution comprised of an 80/17.5/2.5 *v*/*v* proportion of methanol/water/acetic acid with 4 mM ascorbic acid and Resveratrol-14C6 as an internal standard was added to the tube containing the samples, and shaken until the tissue samples were crushed. After shaking, the supernatant was transferred to another tube, and a second extraction was performed using another 250 mL aliquot of extraction solution. The resulting supernatants were pooled and dried under nitrogen, and then reconstituted in 200 mL of 50:50 acetonitrile and water solution containing 5 mM ammonium acetate. The extracts of the blank tissue samples were used to generate standard curves by spiking known amounts of resveratrol standard. The extracts of the experimental tissue samples were then evaluated based on the standard curve generated as previously described [49].

In brief, mass spectrometry was performed using an ABSciex TripleTOF 5600 (Sciex, Foster City, CA, USA) stocked with an electrospray interface with a 50 μm iD capillary and coupled to an Eksigent μUHPLC (Eksigent, Redwood City, CA, USA). Data processing and acquisition, as well as control of the mass spectrometry instrument was performed using the Analyst TF 1.7 software. The following parameters were utilized: source voltage was set to −4.5 kV and maintained at 350 °C, curtain gas was set at 30 psi, gas one at 23 psi and gas two at 30 psi, and acquisition was performed in negative product ion mode. Precursor ions for resveratrol quantification were set to 227.07–185.05, and 233.08–191.07 for the internal standard. A reversed phase Luna Omega Polar C18 column 1 mm i.d., 1.6 μm particles, 50 mm long (Advance Materials Technology, Wilmington, DE, USA) maintained at 50 °C was used for separation. Samples were injected by loop overfilling into a 5 μL loop. The two minutes LC gradient utilized a mobile phase consisting of solvent A (10 mM NH4 acetate in water) and solvent B (10 mM NH4 acetate in acetonitrile) at a flow rate of 50 μL/min. Sciex MultiQuant software (Framingham, MA, USA) was used to quantify the area under the curve.

### 2.9. Statistical Analysis

Statistical analysis of the recording data was performed in the statistical software program R 2.14.2 (The R Foundation, Vienna, Austria) using the MASS package glm.nb() function. A negative binomial parametric model was used to analyze the units per channel and a binomial model was used to analyze the percentage of active channels. Statistical comparisons were made between the two groups at two time ranges (acute and chronic). The acute time range includes the first three weeks of recordings (days 0–21), and the chronic time range includes the fourth through sixth weeks of recording (days 22–42). For statistical analyses of units per channel, total units detected were used. For analyses of percentage of active channels that are able to record units over the 10 best channels, a binomial value (i.e., 0 for a non-active channel and 1 for an active channel) was used. Treatment group, time range, and the interactions between the treatments and time range (i.e., treatment group * time range) were used as terms in the model. Significance was established at *p* < 0.05.

Statistical analysis of histology was done in statistical software program Minitab 19 (Minitab, LLC, State College, PA), using a general linear model with ANOVA to compare the normalized intensity of each concentric bin (50 µm distance intervals from the implanted probe) for the groups. Each ANOVA had a pairwise comparison using a Tukey test with Bonferroni correction, where a *p*-value less than 0.05 was considered significant.

Mass spectrometry results of resveratrol concentrations were statistically analyzed using Minitab 19, using a general linear model with ANOVA to compare the concentrations of resveratrol over time points. A pairwise Tukey test was used for each ANOVA, where a *p*-value less than 0.05 was considered significant.

## 3. Results

### 3.1. Neural Recording Performance

#### 3.1.1. Total Number of Single Units Detected Per Treatment Group

**Figure 2:**
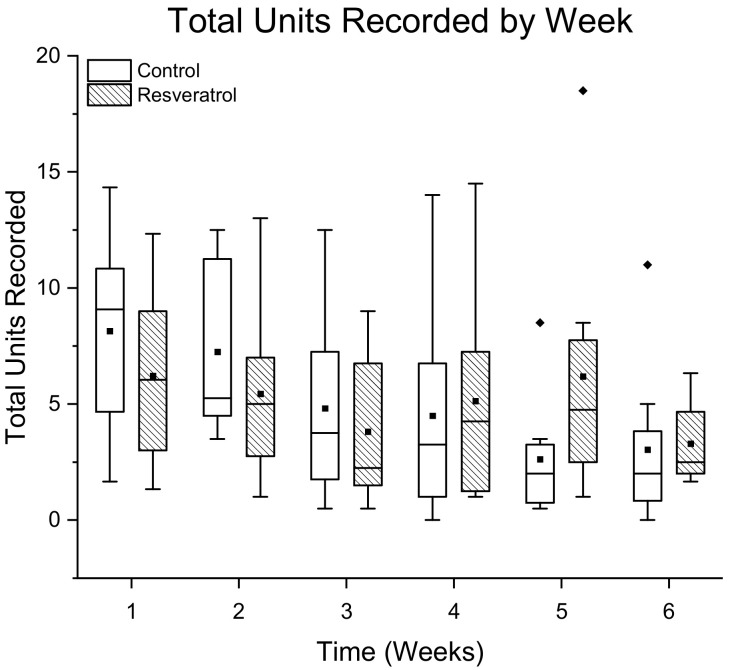
Recording performance of the number of total single units detected in animals administered resveratrol via intraventricular delivery (dashed box) compared to control animals (white box) by week. The total units recorded in the resveratrol group were not significantly different than the controls when compared week by week. The interaction term was significant, indicating a higher slope for the resveratrol group compared to the control group. N = 8 animals per group with 10 channels per animal. The whiskers represent the range within 1.5 IQR, the lines inside the boxes represent the median line, the solid squares in the middle represent the mean and the diamonds represent outliers.

The total number of units detected in each treatment group is displayed as the sum ± standard error across all ten channels for all of the resveratrol (N = 8 animals) and control groups (N = 8 animals) respectively within each time point (Figure 2). The total units recorded each week in the resveratrol group were compared to the total units recorded in the corresponding control group week, but no significant differences were found (Figure 2). A negative binomial linear mix model showed that the interaction term for the resveratrol group was significant (*p* = 0.05), indicating a significantly higher slope for the resveratrol group compared to the control group.

#### 3.1.2. Percentage of Channels Detecting Single Units

**Figure 3:**
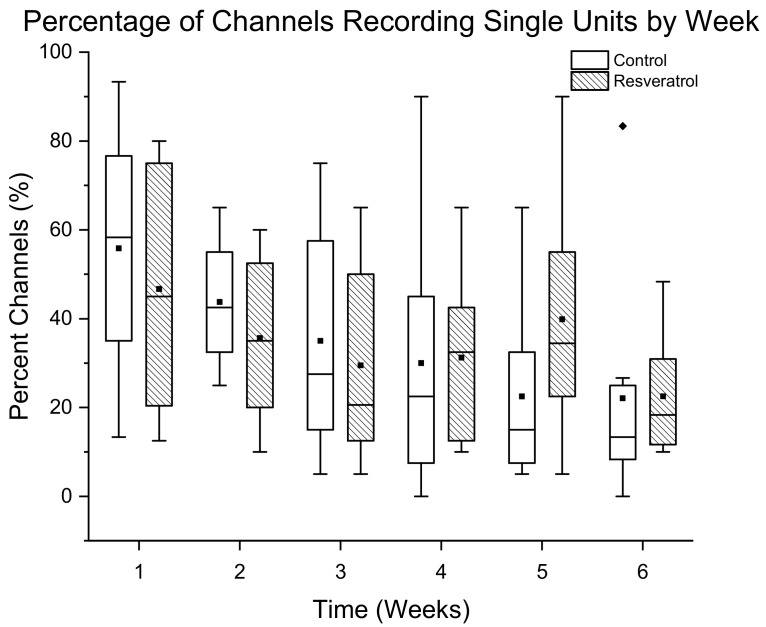
Assessment of recording performance defined as the percentage of channels detecting single units in animals administered resveratrol via intraventricular delivery (dashed box) compared to control animals (white box). No significant differences in the percentage of channels recording single units was seen for the duration of this study. N = 8 animals per group. The whiskers represent the range within 1.5 IQR, the lines inside the boxes represent the median line, the solid squares in the middle represent the mean and the diamonds represent outliers.

The percentage of channels detecting single units is displayed as mean ± standard error for resveratrol (N = 8) and control rats (N = 8) over a 6 week time range (Figure 3). There was no significant difference in the number of channels recording single units. The binomial generalized linear mix model did not show a significant difference between the resveratrol group and the control group at any time points.

#### 3.1.3. Signal and Noise Amplitudes

**Figure 4:**
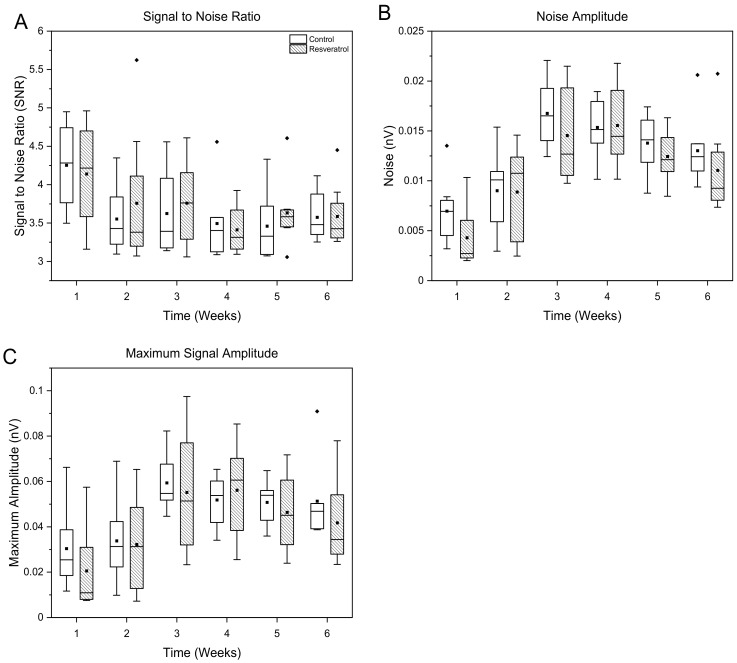
Assessment of recording performance defined as the (**A**) signal to noise ratio, (**B**) noise amplitude, and (**C**) maximum signal amplitude of animals administered resveratrol via intraventricular delivery (dashed box) compared to control animals (white box). No significant differences were seen for the duration of this study, here grouped as acute versus chronic for space and clarity. N = 8 animals per group. The whiskers represent the range within 1.5 IQR, the lines inside the boxes represent the median line, the solid squares in the middle represent the mean and the diamonds represent outliers.

There was no statistical difference found in the signal amplitude, background noise amplitude, or signal to noise ratio between the control and resveratrol groups at any time point (Figure 4).

### 3.2. Evaluation of Tissue Response and Tissue Damage

#### 3.2.1. Oxidative Stress as Measured through Protein Damage

Oxidative stress is characterized as an imbalance of ROS/RNS, which leads to oxidation or peroxidation of lipids, DNA, and proteins, ultimately causing cell death [50,51]. Therefore, oxidative stress was quantified by measuring oxidized proteins. The histological stain for determining the amount of oxidized proteins used here was the modification of the tyrosine residues to their oxidized form, nitrotyrosine.

**Figure 5:**
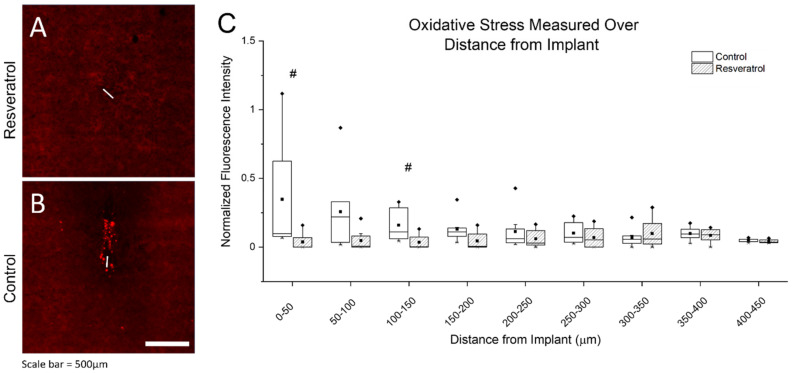
Oxidative stress as measured through protein damage. Representative image of oxidative stress as depicted by oxidized proteins (**A**,**B**), and normalized fluorescence intensity of analyzed images in bins of 50 mm distances from the electrode implant tract (**C**). There was a significant (# *p* < 0.05) decrease in nitrotyrosine detected in the resveratrol group compared to the control group at the 0–50 μm and 100–150 μm ranges. The white line depicts the electrode implantation site. The whiskers represent the range within 1.5 IQR, the lines inside the boxes represent the median line, the solid squares in the middle represent the mean and the diamonds represent outliers.

The resveratrol group had significantly less nitrotyrosine than the control group, shown in Figure 5. Specifically, quantification of the expression of nitrotyrosine indicated higher expression of nitrotyrosine in the control group compared to the resveratrol group over both the 0–50 mm and 100–150 mm radial distances from the electrode implantation site. Figure 5A,B displays representative images.

#### 3.2.2. Activation of Microglia, Macrophage, and Astrocytes, Neuronal Nuclei Density, and Blood–Brain Barrier Permeability

**Figure 6:**
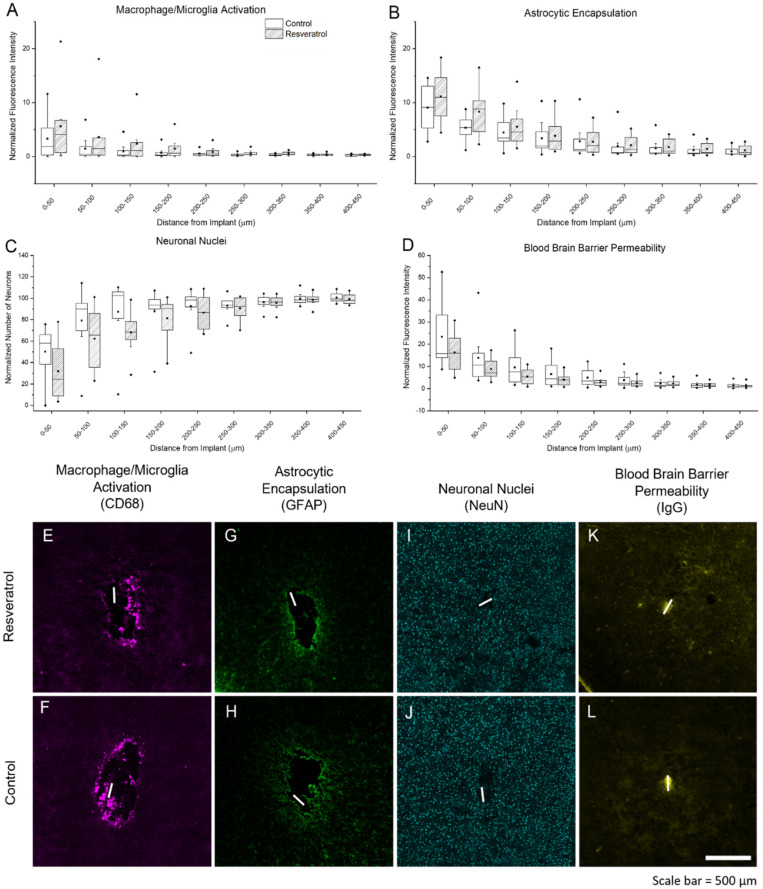
Activation of microglia, macrophage, and astrocytes, neuronal nuclei density, and blood–brain barrier permeability. Fluorescent intensity analysis plots and representative images of activated microglia/macrophage (CD68) (**A**,**E**,**F**), astrocytes forming the glial scar (GFAP) (**B**,**G**,**H**), and blood–brain barrier permeability (IgG) (**D**,**K**,**L**), in bins of 50 µm width from the electrode implant tract. Neuronal nuclei density (NeuN) (**C**,**I**,**J**) was determined by counting neurons in 50 μm wide bins from the electrode implant tract. The white line depicts the electrode implantation site. The whiskers on the graphs represent the range within 1.5 IQR, the lines inside the boxes represent the median line, the solid squares in the middle represent the mean and the diamonds represent outliers.

The documented mechanisms by which resveratrol provides antioxidative relief includes preventing the production of new ROS/RNS, scavenging existing ROS/RNS, and upregulating the expression of endogenous enzymes such as SOD and catalase [13,52]. Glial cell activation has been found to be more important in the acute stages of the inflammatory response, while endogenous enzyme upregulation is more important at chronic stages, and scavenging of free ROS/RNS occurs throughout both time points [53,54,55]. Here, we used cluster of differentiation 68 (CD68 transmembrane glycoprotein) and glial fibrillary acidic protein (GFAP intermediate filament protein) to look at the changes in expression levels of microglia/macrophages and astrocytes, respectively. Histological markers for immune cell and glial cell activation showed no significant differences between control and resveratrol treated groups (Figure 6A,B,E–H).

Histological detection of neurons through the quantification of cells staining positive for neuronal nuclei (NeuN) is a standard in this field [23,56,57,58]. Quantification of neuronal nuclei density (Figure 6C,I,J) also showed no significant different between the resveratrol group and control group.

Finally, blood–brain permeability has been used as a benchmark for brain tissue health, as an intact blood–brain barrier is important in regulating the flow of nutrients to cell, among other functions [59,60]. Here, we determined blood–brain barrier permeability by staining for the presence of IgG, which are normally not found commonly in the brain parenchyma, but increase when the blood–brain barrier is breached or leaky [61,62]. The results showed that there are no observed differences in blood–brain barrier permeability (Figure 6D,K–L) between the resveratrol-treated and control groups. Figure 6 illustrates representative images and plots of all of the aforementioned histological stains.

### 3.3. Bioavailability of Resveratrol

A concern at the onset of the study was the possible effect of the foreign body response to the implanted osmotic pump slowing or preventing the delivery rate of resveratrol with time. Therefore, an additional cohort of animals (N = 4) was implanted with non-functional ‘dummy’ probes and osmotic pumps delivering resveratrol, to validate the in vivo performance and delivery rates of the osmotic pumps.

#### 3.3.1. Validation of In Vivo Delivery Rate

**Figure 7:**
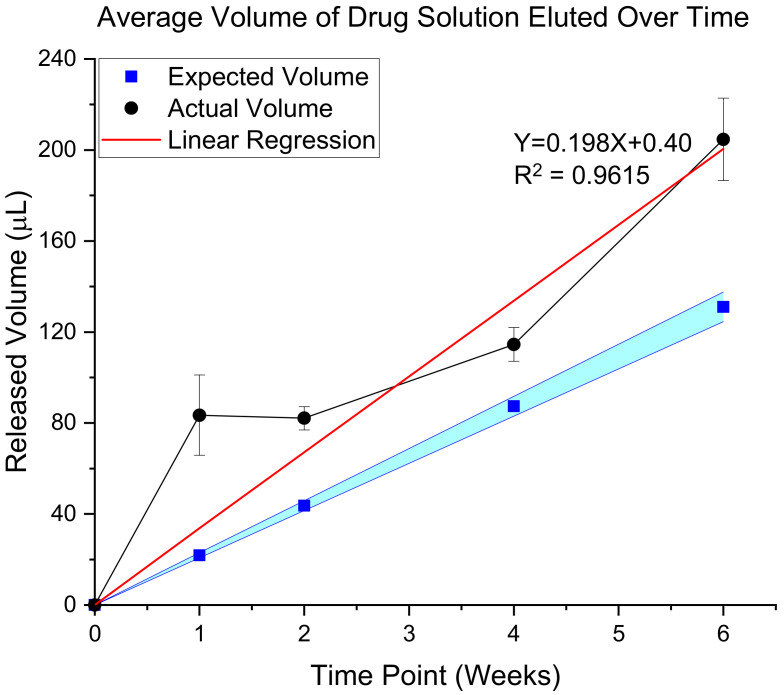
Validation of In Vivo Delivery Rate. The predicted flow rate (black line, circle markers) for osmotic pumps is plotted with the actual flow rate (blue squares in cyan band) for N = 4 animals. A linear equation was fit to the measured data (red line), showing that the actual flow rate was 19.8 μL/h with an R2 value of 0.9615. The expected flow rate is 13 μL/h, plotted with a +/− 5% band, the expected acceptable error of the osmotic pump flow rates.

To create a temporal understanding of actual versus predicted flow rates, animals were euthanized at additional time points (1, 2, 4, and 6 weeks post-implantation). Quantification of the amount of solution left in the pumps upon extraction, compared to the pre-implantation volume allowed us to generate an understanding of the flow rates during use (Figure 7). Interestingly, our results indicated that the flow rate of resveratrol from the osmotic pumps was slightly higher (19.8 mL/h) flow rate than the expected rate (13 mL/h) (Figure 7). Although the R2 value seems to be high at 96.2%, indicating steady elution, closer observation of the data points suggests a rapid elution during the first week, followed by a plateaued release through week 4, but then another rapid elution at week 6. To understand the possible explanations for the release, the explanted cannulas were investigated and imaged.

**Figure 8:**
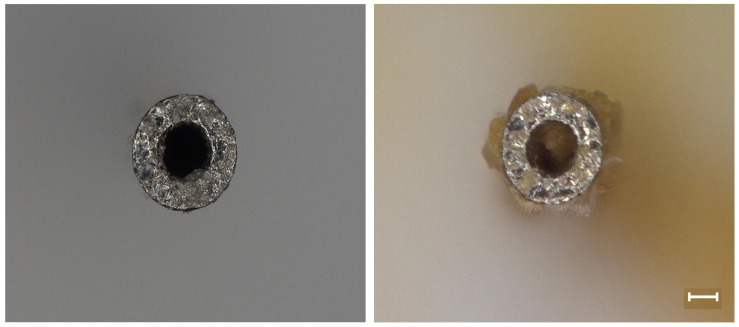
High Magnification Image of Explanted Cannula. The explanted cannulas seemed to show cellular debris and brain tissue on the inside of the cannulas, which may have caused the unstable drug delivery rate. Scale bar = 100 μm.

Investigation of the explanted cannulas showed that some of the cannulas were clogged with possible brain tissue or cellular debris (Figure 8), which could explain the observed variability in drug elution rates. Additionally, histological analysis of the material inside of the cannulas by staining for cellular content with DAPI showed cellular nuclei (data not shown), indicating that the matter clogging the inside of the cannulas were cellular.

#### 3.3.2. Quantification of Resveratrol in Tissue

In order to determine the efficiency and effectiveness of intraventricular delivery of resveratrol, an understanding of the amount of resveratrol in the area around the site of implantation is necessary. Intraventricular delivery is an attractive method of delivery because it bypasses the difficulties of first pass metabolism and the blood–brain barrier. However, the drug must still diffuse through the brain tissue to the site of implantation to be effective. To assess resveratrol diffusion to the electrode, tissue samples from the implant area, an area directly contralateral to the implant, and the liver were collected at several time points ranging from 1 to 6 weeks of continuous intraventricular drug delivery (N = 4).

**Figure 9:**
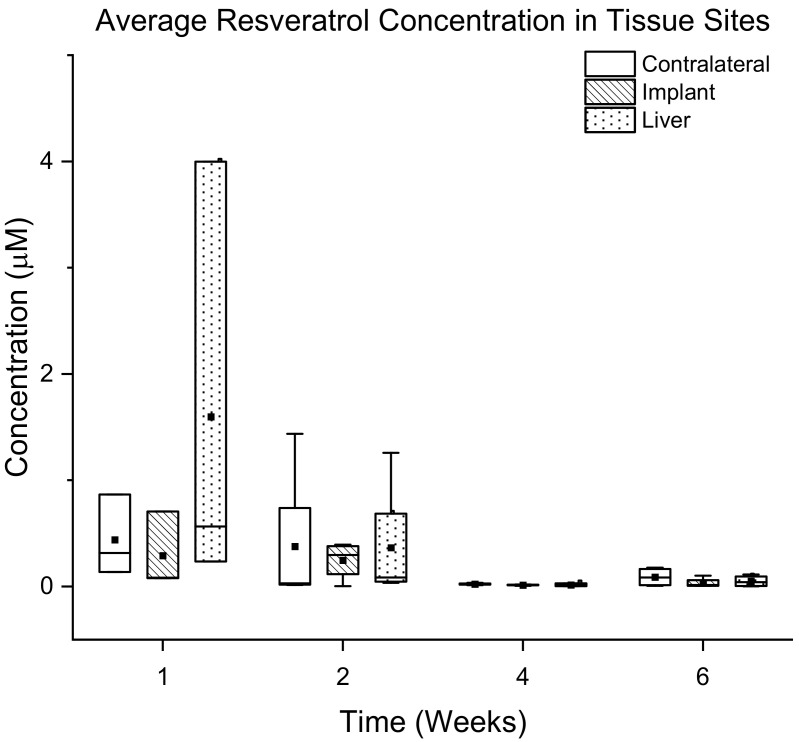
Quantification of Resveratrol in Tissue Over Time. Average concentration and standard error in µM of resveratrol at the implant site, in brain tissue contralateral to the implant, and in the liver. There is no significant difference in resveratrol levels at all time points, but there is a general trend of higher levels at the acute time points (1 and 2 weeks), and a decrease at chronic time points (4 and 6 weeks).

Free circulation of cerebrospinal fluid is expected between all ventricles, inner spinal cord and the spaces between the brain and spine. Free circulation should allow for high concentrations of resveratrol in both hemispheres of the brain. Collected tissue was digested and resveratrol was extracted as described above. Isolated resveratrol was quantified via mass spectrometry analysis of harvested tissue from animals at different time points. Analysis showed that the level of resveratrol at all three tissue sites for the acute time points (1 and 2 weeks) were not statistically significantly different compared to chronic time points (4 and 6 weeks). However, there is a general trend of decreased resveratrol levels at all tissue sites from 2 weeks to 4 weeks (Figure 9). Initial data from mass spectrometry presented the data in units of ng of resveratrol per gram of brain tissue. For ease of understanding, these concentration values have been converted into µM resveratrol, using a density of rat brain tissue of 1.05 g/mL and density of liver tissue of 1.07 g/mL [63,64].

## 4. Discussion

Applications for implanted intracortical microelectrodes offer the potential to drastically improve the quality of life for patients as well as provide a critical means to further basic science discoveries [2,10,11,65]. Unfortunately, intracortical microelectrodes have a documented gradual temporal decline in recording performance, thus limiting their long-term functional use [66,67,68,69]. ROS/RNS generation as a result of the foreign body response can cause material breakdown of the microelectrodes, and is thought to contribute to “dieback” of neurons close to the microelectrode recording sites [10,14,18,20,68].

Antioxidants have the ability to directly neutralize ROS/RNS and increase antioxidative enzyme synthesis, and have thus emerged as a powerful tool for preventing oxidative stress [70,71]. For example, in Alzheimer’s and Parkinson’s diseases, natural phenols and “food-derived” antioxidants have shown significant promise [72,73]. Success of antioxidants (e.g., resveratrol, quercetin, curcumin, melatonin, vitamins C and E) is likely due to their ability to directly neutralize ROS, and their ability to freely penetrate cells [74]. Therefore, we have explored the use of several antioxidants in preventing microelectrode-induced oxidative stress [13,35,70,75]. Unfortunately, due to poor solubility in saline, short half-life in sera, and low bioavailability due to extensive first pass metabolism and the blood–brain barrier, delivery to the brain in therapeutic concentrations has proven to be a major hurdle [13,29,35,36,38,76]. One antioxidant that is of particular interest to our group is resveratrol. Resveratrol works to decrease ROS/RNS by quenching pre-existing ROS/RNS and preventing the generation of new ROS/RNS [13,54]. Therefore, the goal of this study was to investigate the applicability of ventricular delivery of resveratrol to the microelectrode tissue interface to circumvent bioavailability hurdles of resveratrol delivery to the brain. We also sought to assess the efficacy of intraventricular cannula delivery of resveratrol in a PEG200 solution to improve the recording performance of intracortical microelectrodes. Furthermore, we examined the tissue adjacent the microelectrode for markers of oxidative stress and neuroinflammation, while measuring the accumulation of resveratrol within brain and liver tissue as a method of determining delivery efficacy. The osmotic pumps used in the current study were only rated for 6 weeks of delivery. Therefore, the most chronic time point investigated here was 6 weeks to not confound our results with the addition of a revision surgery to replace the pumps.

Here, we investigated the neuroinflammatory response to the microelectrodes in animals treated with resveratrol compared to non-treated control animals (Figure 4 and Figure 5). Six weeks post-implantation has been considered just within the timeframe of the chronic stage of the neuroinflammatory response to intracortical microelectrodes [22,77]. We saw no significant differences in the activation of macrophages, microglia, or astrocytes (Figure 6), indicating no significant improvements in neuroinflammation mitigation. Resveratrol-treated animals displayed a significant reduction in the formation of nitrotyrosine (Figure 5). Tyrosine residues are prone to ‘attack’ by ROS/RNS, leading to the formation of nitrotyrosine. Once oxidized, proteins are unable to function correctly, if at all, severely impeding metabolic processes such as those that support maintenance of healthy neurons [78,79]. However, it is important to note that polyethylene glycol (PEG) may also have antioxidative properties, which may have led to confounding results [80,81,82].

Delivery of resveratrol to the brain at therapeutic levels has remained difficult since we began pursuing it in 2010 [13,35,75]. In the current study, isolation of resveratrol and detection with mass spectrometry analysis showed that levels of resveratrol are not significantly different between all of the time points. However, there is a general trend where levels of resveratrol drastically decrease at more chronic time points (4 and 6 weeks) around the implant site, in the contralateral hemisphere and in the liver (Figure 9). Recording analysis also showed that there was no significant difference between the resveratrol and control groups in total units detected and percentage of channels recording single units. Analysis of the explanted osmotic pumps showed a linear rate of elution with a 96.2% R2 value (Figure 7). However, the trend of the rates showed that the rate of drug elution from the pumps plateaued after week 4, but increased at 6 weeks. Although there was an increased release of resveratrol at 6 weeks observed from the explanted pumps, this was not demonstrated in the mass spectrometry analysis of resveratrol concentration around the implant site. Together, these results indicate that perhaps the majority of the eluted drug at 6 weeks was stuck in the tubing and cannula portion of the device due to the clogging of the cannula, making it difficult for the full amount of drug to reach the brain tissue. Conceivably there is an inconsistent delivery of resveratrol that was confounded by unexpected factors, such as cellular ingrowth or physical blockage of the cannulas by brain tissue during the implantation process. Further analysis of the explanted cannulas indeed showed cellular matter inside of the cannulas that could have blocked consistent drug delivery (Figure 8), likely contributing to the variable drug elution rates, tissue response, and recording performance.

Our results demonstrated no improvement in inflammation, but significant decrease in oxidative stress, decreasing tissue resveratrol concentrations over time, and no electrophysiological improvements. Collectively, these results suggest that while intraventricular delivery of resveratrol allowed for some drug to reach the effected tissue, it is likely not the optimal drug delivery approach to deliver resveratrol (or other molecules) to the intracortical microelectrode/tissue interface. Quantification of resveratrol concentrations around the implant site revealed nano-molar concentrations of resveratrol at the tissue adjacent to the implant. Previous in vitro studies suggest micro molar ranges (~25 µM) of resveratrol are required for neuroprotection [83]. Such low levels in the brain tissue could be due to poor aqueous solubility of resveratrol, cannula obstruction due to brain or cellular matter, or inconsistent elution of drug solution from the pumps. In fact, resveratrol can precipitate out of the solution once it reaches the cerebrospinal fluid, consequently adhering to lipids, and diffusing through brain tissue as a result of its poor solubility [36,37,38]. Additionally, it was observed that the explanted cannulas appear to have brain tissue or cellular debris inside of them which may have been introduced during the implantation surgery. Future work can avoid this concern by utilizing a dissolvable cover at the end of the cannula or even a completely different cannula design that does not implement the outlet port on the tip, rather on the sides. Elution rates were also not consistent with expected rates and were outside of the admissible 5% error band of elution rate for several of the osmotic pumps. Therefore, future efforts to develop novel microelectrode designs should enable more local and precise delivery of resveratrol, such as microfluidic probes or chronic drug-eluting probes [39,84,85]. Microfluidic probes indeed seem to be a logical next step in our work, whereby resolving the issue of reliable, prolonged drug delivery directly at the site of implantation by connecting a microfluidic probe to an osmotic pump. Other groups have explored fabrication methods and materials in order to achieve such a device by integrating microfluidic channels onto functional silicon probes and flexible polymers [86,87,88]. Our lab has also explored microfluidic probe fabrication onto mechanically adaptive polymers in hopes of reducing inflammation via both drug delivery and decreased differential strain introduced to the brain-probe interface with stiff silicone probes [89]. Polymer probes with integrated microfluidic channels have been studied on their own, but successful chronic studies using such probes have been hindered by the ability to implant without buckling and degrading. Such methods may improve the longevity and quality for intracortical microelectrode recordings and help to study additional effects resveratrol may have on not only ROS/RNS production and quenching, but also on other systems that may affect brain tissue health and/or prevent electrode materials degradation.

## 5. Conclusions

The current study has implemented intraventricular delivery of resveratrol in an attempt to circumvent the bioavailability hurdles of resveratrol delivery to the brain, while also investigating the applicability to improve both the electrode recording performance and the tissue response. Results indicated that there were no significant differences in electrophysiological recording performance between the groups receiving resveratrol through intraventricular cannulation compared to control groups when assessed weekly over the six-week study. Interestingly, there were significantly more total units detected with the resveratrol group compared to the controls during the chronic time points, when the time points were grouped as acute (weeks 1–3) and chronic (weeks 4–6). Histological analysis showed a significant reduction in tissue oxidation with the intraventricular resveratrol delivery group compared to the control group, but there was no statistical difference detected in the other biomarkers of inflammation such as blood–brain barrier permeability, activated microglia/macrophages, astrogliosis, and neuronal nuclei density. Further investigation into the drug elution rates showed that intraventricular delivery was not consistent, and only nanomolar concentrations of resveratrol were reaching the brain. Collectively, the results presented here show that intraventricular delivery of resveratrol may not be ideal due to confounding factors such as cannula clogging, inconsistent elution rates, and precipitation of resveratrol when introduced to CSF. A more tunable method for local delivery that is capable of responding to metabolic fluctuations may allow for more controlled resveratrol concentrations in long-term applications.

## Data Availability

Data available on request due to privacy/ethical restrictions.
